# Vehicle Pose Measurement with a Single Image via the Double-Dual-Ellipse Feature of Wheel Rims

**DOI:** 10.3390/s26185880

**Published:** 2026-09-17

**Authors:** Guan Xu, Rui Wang, Huiying Lin

**Affiliations:** Department of Vehicle Operation Engineering, Transportation College, Nanling Campus, Jilin University, Changchun 130025, China; xuguan@jlu.edu.cn

**Keywords:** vehicle pose measurement, single-image, double-dual-ellipse feature, vehicle geometry, verification

## Abstract

The precise measurement of the vehicle pose is a key technology for achieving the intelligent inspection and understanding of vehicle behavior. This paper presents a vehicle pose measurement method utilizing a line-space dual-ellipse representation of wheel rims. First, a dual quadratic curve formulation is established to model wheel-rim projection ellipses, where the common tangent extraction is transformed into a generalized eigenvalue problem, enabling analytical vehicle rotation recovery from a single image. Second, a metric vehicle translation recovery scheme is developed by integrating the vehicle’s longitudinal wheelbase constraint. The research conducts comprehensive and systematic experimental verification, including simulation, comparative analysis, vehicle test, and the physical benchmark experiment. The experimental results demonstrate that the proposed method achieves high measurement accuracy and reliability, providing a solution for vehicle pose measurement in practical intelligent vehicle inspection.

## 1. Introduction

Object pose detection, which estimates the position and orientation of an object in 3D space, often referred to as 6 degrees of freedom (DOF) pose [1], is a core research topic in industrial fields such as industrial automation [2,3], robotics [4], vehicle inspection [5,6], and geographic information [7,8]. Vehicle pose measurement, which accurately acquires the position and orientation, represented by 3-DOF rotation and 3-DOF translation of a vehicle in 3D space through sensor data, is key for autonomous driving [9] and intelligent transportation systems.

Vehicle pose estimation methods are generally classified into two categories. The first category relies on sensor fusion technology, combining data from LiDAR and multiple cameras [10,11]. A portable, non-contact pose measurement system with a long detection distance was proposed by Kim et al. [12]. The system is based on 1-D laser sensors and a camera. The system can detect 6 DOF without the initial posture of the object and prior knowledge of the object. Ni et al. [13] proposed an RSU-based vehicle pose detection method that integrates onboard GPS positioning, roadside camera-based vehicle detection and tracking, and OBU–RSU communication to obtain vehicle pose information in real time. Although effective, multi-sensor systems have issues such as complex calibration, high hardware costs, and large computational overhead, limiting their widespread application in cost-sensitive scenarios.

In contrast, vision-based methods have become an attractive alternative due to the low cost, low power consumption, simple system structure, and sufficient information [14]. Moreover, the camera is a passive device that fundamentally avoids the problem of same-frequency interference on multi-sensor platforms. However, the 3D pose estimation from a single 2D image is an inherently ill-posed problem, mainly due to the loss of depth information and the ambiguity caused by the perspective projection [15,16]. To address this issue, pose measurement methods primarily fall into two categories: data-driven learning methods and geometric model-driven methods.

Data-driven methods first regress the projection points of the 3D bounding box of the vehicle on the 2D image through a convolutional neural network (CNN). Then the pose is solved through the perspective *n*-point algorithm (P*n*P) [17]. Song et al. [18] first introduced this paradigm for vehicle pose estimation. The method first regresses the projection coordinates of the 8 corner points of the predefined 3D bounding box of the vehicle on the 2D image through a CNN. Then the predicted 2D-3D point correspondences are used and combined with the geometric P*n*P to calculate the 6-DOF pose of the vehicle. Although deep learning-based methods have performed well in many tasks, the performance heavily relies on the accuracy of the keypoint detection. Furthermore, pre-training with large-scale high-quality labeled data incurs high training costs and may result in insufficient generalization [19,20].

Vision-based methods based on geometric models provide a distinct solution path. Instead of learning patterns from massive data, the methods directly utilize the geometric principles of imaging to solve for the pose, naturally avoiding pre-training processes and dependence on datasets. During the projection process, the transformation of the 3D point into the 2D representation inherently results in the loss of a DOF that is essential for pose estimation. Consequently, supplementary information is required to compensate for this loss and fully recover the missing DOF. Stereo vision or binocular vision methods adopt more views to supplement the lost DOF information. Therefore, the 3D information and spatial pose of the object can be obtained from the multiple views. Duran [21] et al. proposed a stereo vision system for inter-vehicle distance measurement. Stereo image pairs acquired at predefined distances are employed to train a neural network, which subsequently generates real-time distance estimations to enhance driver assistance. In the bionic binocular system for robotics, Fan et al. [22] actively aligned the optical axes of the two cameras toward a common 3D point using a servo mechanism. This configuration thereby minimizes the impact of extrinsic parameters. Zhao et al. [23] proposed a multi-camera system for rigid body 6-DOF measurement that is adaptable to objects of diverse geometries. The method begins by determining the relative poses between the cameras and then establishes a measurement model based on a set of manually marked points on the rigid body. Finally, the precise pose of the target object is computed via the least squares method. A vision-based 6-DOF pose measurement method was proposed by Kim et al. [24] for the pose evaluation of Stewart platforms and robots. It employs a phase-encoding strategy on fixed target patterns, enabling high-precision evaluation by computing absolute pose data directly from captured images. However, the 3D pose estimation from a single 2D image is an inherently ambiguous problem, and its accuracy and robustness depend heavily on the stability and measurability of the selected visual features. In terms of irregularity detection of vehicle wheels and rims, Y. Wiseman’s work [25] demonstrates how JPEG images of vehicle tires, captured by a standard digital camera, already contain sufficient high-frequency information to detect structural damage or irregularities on the very wheel rims that the present dual-ellipse pose estimator relies upon. This lightweight compressed-domain check would allow the single-image pipeline both to recover 6-DOF vehicle pose and simultaneously to flag compromised rim contours, thereby turning the geometric measurement system into a more comprehensive intelligent-inspection tool that jointly addresses pose accuracy and tire-safety monitoring.

In response to the current situation where the pose measurements rely heavily on multi-camera systems or multi-position observations with a single camera, this study observes that the wheel rims on both sides of a vehicle have symmetry and structural stability, and can be adopted as ideal feature targets in the visual measurement. Moreover, different vehicle poses result in different projected elliptical shapes of the two wheel rims in the image plane, and their common tangent positions also change accordingly. Therefore, the projected ellipses of the two wheel rims contain rich vehicle pose information. The traditional method for solving common tangents of ellipses based on the point space is computationally complex and susceptible to noise interference. However, in the dual line space, the common tangents of two ellipses can be likened to the intersections of two ellipses in the point space. This idea not only simplifies the description of geometric relationships, but also lays the foundation for constructing numerically stable and analytically solvable mathematical models. According to the above ideas, the main contributions of the article in vehicle pose measurement are as follows:

1. A dual-line-space formulation based on the double-dual-ellipse feature of vehicle wheel rims is established to parameterize the geometric feature of the vehicle. An analytical solution for vehicle rotation is achieved from a single image.

2. A vehicle metric translation recovery scheme is developed by integrating the longitudinal wheelbase constraint of the vehicle. This approach completes the closed-form 6-DOF measurement in a single image for vehicle inspection.

3. The simulation experiments, comparative experiments, vehicle experiments, and physical benchmark verification experiments have been conducted for this method. The robustness and reliability of the method have been verified in real scenarios.

The remainder of this paper is organized as follows. Section 2 explains the principle of the vehicle pose measurement with a single image (SI) and the double-dual-ellipse feature of wheel rims. Section 3 describes the simulations and experiments. Section 4 summarizes the article.

## 2. SI-Line-Space Measurement Method

### 2.1. Vehicle Pose Measurement Principle

**Figure 1:**
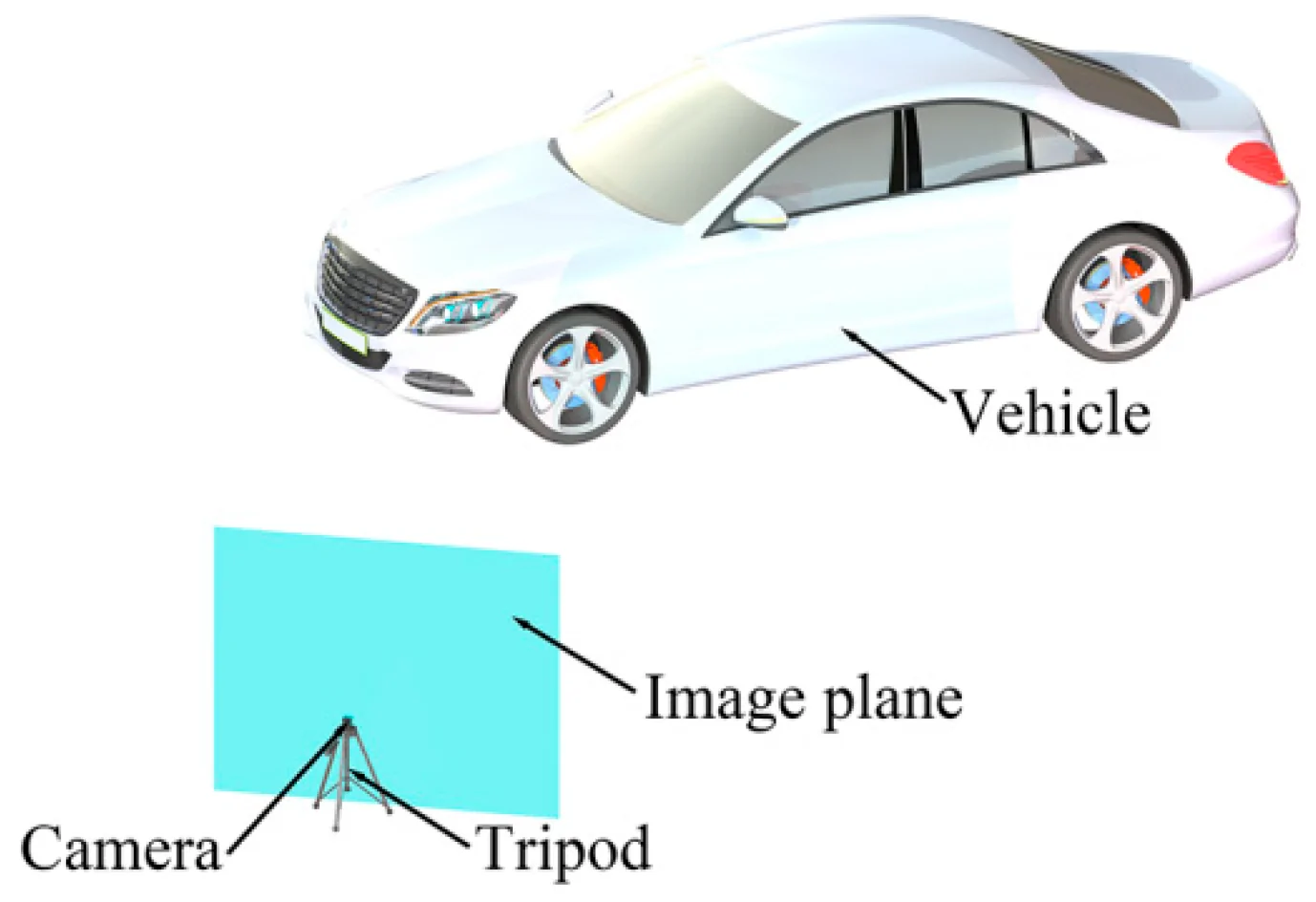
Vehicle pose measurement with the double-dual-ellipse feature of wheel rims.

**Figure 2:**
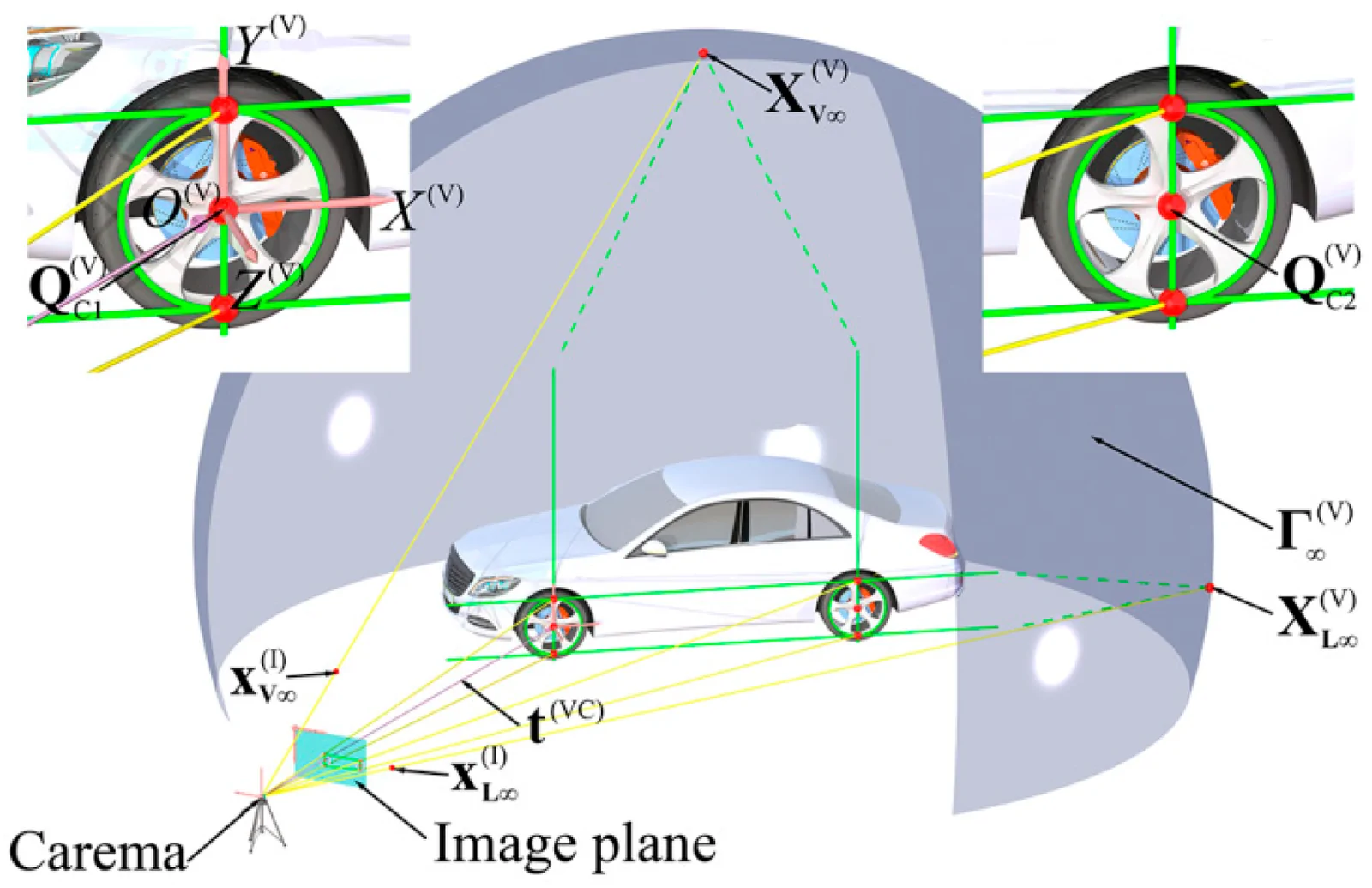
Generation process of double-dual-ellipse feature of wheel rims.

**Figure 3:**
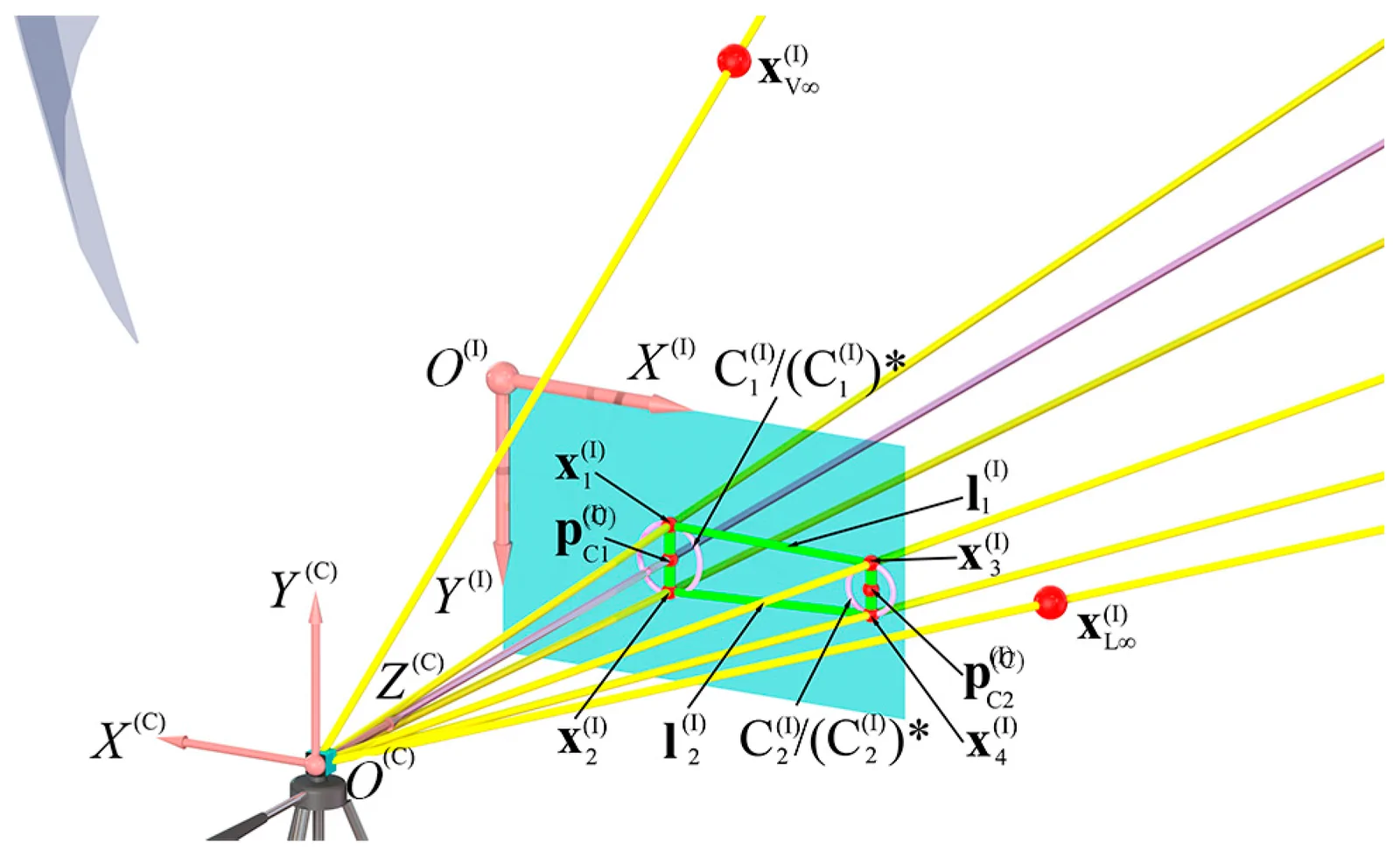
Double-dual-ellipse feature of wheel rims.

Due to the implicit representation of the pose information of the vehicle at different positions by the two wheel rims, a measurement system as shown in Figure 1 is constructed to deeply reveal the inherent relationship between the double-dual-ellipse feature of wheel rims and the vehicle pose information, achieving the SI measurement of the vehicle pose. The generation process of the double-dual-ellipse feature of wheel rims is shown in Figure 2 and the double-dual-ellipse feature of wheel rims is shown in Figure 3. The coordinate system of the camera and the image coordinate system are *O*^(C)^-*X*^(C)^*Y*^(C)^*Z*^(C)^ and *O*^(I)^-*X*^(I)^*Y*^(I)^. The coordinate system of the vehicle is *O*^(V)^-*X*^(V)^*Y*^(V)^*Z*^(V)^. Superscripts (I), (C), and (V) denote variables expressed in the image, camera, and vehicle coordinate systems, respectively. The superscript * denotes the dual conic representation. The origin *O*^(V)^ is the center of the front wheel rim. The *O*^(V)^-*X*^(V)^ axis is along the line connecting the centers of the front and rear wheel rims. The *O*^(V)^-*Z*^(V)^ axis is perpendicular to the front wheel rim. The image of the vehicle is captured by the camera and the wheel rim circles are projected to ellipses on the image plane. Therefore, the double-dual-ellips feature is generated from the projected ellipses of two wheel rim circles. The feature uniformly describes the geometric constraints of two ellipses in the dual line space, which not only simplifies the mathematical model for the vehicle rotation, but also provides a key geometric basis for the stable and closed-form solution of the vehicle’s 6 DOF. Based on the projection information of the double-dual-ellipse feature in a SI, an analytical measurement model is established to achieve accurate vehicle pose detection. The list of abbreviations is shown in Table 1.

### 2.2. SI-Line-Space Determination of Vehicle Direction

As the intersection point of the common tangent lines of the two projected ellipses of wheel rims in the image implies the direction of the vehicle body, it is important to accurately solve the common tangent lines of the two ellipses. The wheel rim ellipse detected in the image [26] is essentially a collection of its contour tangents in the dual line space. As the key to vehicle pose measurement lies in extracting the common tangent between two ellipses, the ellipses based on the dual quadratic curves in the dual line space are adopted to solve the common tangent. Due to the dual forms of ellipses directly describing the tangent distribution of ellipses in the line space, they naturally fit with the common tangent to be solved. Therefore, in Figure 3, the symmetric matrices $C_{1}^{\left(I\right)}$ and $C_{2}^{\left(I\right)}$ of the two projection ellipses of the wheel rims in the *O*^(I)^-*X*^(I)^*Y*^(I)^ plane are represented by the dual quadratic curve form as [27]

(1) $$\left\{\begin{array}{c} {\left(l_{i}^{\left(I\right)}\right)}^{T}{C_{1}^{*}}^{\left(I\right)}l_{i}^{\left(I\right)}=0\\ {\left(l_{i}^{\left(I\right)}\right)}^{T}{C_{2}^{*}}^{\left(I\right)}l_{i}^{\left(I\right)}=0 \end{array}\right.$$

where the matrices of the dual quadratic curves are ${C_{1}^{*}}^{\left(I\right)}=[\det(C_{1}^{\left(I\right)})]{\left(C_{1}^{\left(I\right)}\right)}^{-1}$ and ${C_{2}^{*}}^{\left(I\right)}=[\det(C_{2}^{\left(I\right)})]{\left(C_{2}^{\left(I\right)}\right)}^{-1}$.

The generalized eigenvalue equation constructed by the Lagrange multiplier method is a robust strategy for the intersection problem of dual quadratic curves [27]. The parameterized configuration of vehicles is implemented to address the unique spatial symmetry and longitudinal wheelbase constraints of vehicle wheels. A specialized constraint-solving model for vehicle features is constructed, as shown in Equations (2) and (3). As $l_{i}^{\left(I\right)}$, *i* = 1, 2, is the common tangent of the two projection ellipses of two wheel rims, it simultaneously satisfies the aforementioned Equation (1). In order to effectively extract this geometric feature that can characterize the orientation of the vehicle, this paper fully utilizes the coplanarity and spatial symmetry of the vehicle wheel rims to construct a Lagrangian solution model. The first term in Equation (1) is adopted as the objective function and the second term in Equation (1) is considered as a constraint.

(2) $$\underset{l_{i}^{\left(I\right)}}{\min\;}\{{\left(l_{i}^{\left(I\right)}\right)}^{T}{C_{1}^{*}}^{\left(I\right)}l_{i}^{\left(I\right)}\}\;\;\;\;\;\;\;\;\;\;\;\;\;s.t.\;\;\;{\left(l_{i}^{\left(I\right)}\right)}^{T}{C_{2}^{*}}^{\left(I\right)}l_{i}^{\left(I\right)}=0\;\;\;\;\;\;\;$$

Therefore, based on the geometric tangent constraint of the vehicle wheel rims, the generalized eigenvalue equation for the common tangent $l_{i}^{\left(I\right)}$ can be expressed as

(3) $${C_{1}^{*}}^{\left(I\right)}l_{i}^{\left(I\right)}=\lambda {C_{2}^{*}}^{\left(I\right)}l_{i}^{\left(I\right)}$$

According to Equation (3), the generalized eigenvalue equation for the common tangent line $l_{i}^{\left(I\right)}$ of the vehicle wheel rims can be solved by

(4) $${{\det[C}_{1}^{*}}^{\left(I\right)}-\lambda {C_{2}^{*}}^{\left(I\right)}]=0$$

where **0** = [0, 0, 0]^T^. The uniqueness of the recovered 6-DOF pose requires that the two projected ellipses be non-degenerate and that the matrices ${C_{1}^{*}}^{\left(I\right)}$ and ${C_{2}^{*}}^{\left(I\right)}$ of the corresponding dual conics be non-singular. When the optical axis of the camera is perpendicular to the plane of the rim circle, the projection of the wheel rim becomes a circle. The long and short axes of the ellipse are equal, and the eigenvalues of Equation (4) exhibit multiple roots or symmetry degradation. When the optical axis is parallel to the plane of the wheel circle, the wheel rim projections further degenerate into a straight line, and the corresponding matrices ${C_{1}^{*}}^{\left(I\right)}$ and ${C_{2}^{*}}^{\left(I\right)}$ are not of full rank. By solving the eigenvalue equation, its eigenvectors directly correspond to the projection constraints of the longitudinal axis of the vehicle on the image.

As the intersection point of the two parallel tangent lines of the wheel rims in Figure 2 is an infinite point $X_{L\infty }^{\left(V\right)}={\left[{\left(d_{L}^{\left(V\right)}\right)}^{T}\;\;\;0\right]}^{T}$ on the infinite plane $\Gamma_{\infty }^{\left(V\right)}$ and $d_{L}^{\left(V\right)}$ is the direction vector of the two parallel tangent lines, the infinite point represents the direction of the longitudinal axis *O*^(V)^-*X*^(V)^ of the vehicle. This infinite point fundamentally characterizes the vehicle’s heading orientation. According to the principle of homogeneous coordinates in projective geometry, the projection of the infinite point $X_{L\infty }^{\left(V\right)}$ on the image is solved by

(5) $$x_{L\infty }^{\left(I\right)}=l_{1}^{\left(I\right)}\times l_{2}^{\left(I\right)}$$

According to the perspective projection principle [27] from spatial points to image points, the longitudinal axis direction of the vehicle can be determined by the projection point $x_{L\infty }^{\left(I\right)}$ on the image, as

(6) $$d_{L}^{\left(C\right)}=K^{-1}x_{L\infty }^{\left(I\right)}$$

where K = [*k*_mn_]_3×3_ is the calibrated camera intrinsic matrix.

By the derived tangent lines and the projected wheel ellipse, the precise tangent point can be analytically solved as

(7) $$x_{j}^{\left(I\right)}=\underset{x_{j}^{\left(I\right)}\in C_{1}^{\left(I\right)},C_{2}^{\left(I\right)},\;j=1-4}{\arg\min}|{\left(x_{j}^{\left(I\right)}\right)}^{T}{\left(l_{i}^{\left(I\right)}\right)}_{\mathrm{norm}}|$$

where ${\left(l_{i}^{\left(I\right)}\right)}_{\mathrm{norm}}$ is the normalized tangent line.

Due to the fact that the two lines connecting the tangent points of the wheel rims still intersect at an infinity point $X_{V\infty }^{\left(V\right)}$, in projective geometry, the projection point of the infinity point is

(8) $$x_{V\infty }^{\left(I\right)}=(x_{1}^{\left(I\right)}\times x_{2}^{\left(I\right)})\times (x_{3}^{\left(I\right)}\times x_{4}^{\left(I\right)})$$

According to Equations (6) and (8), the vertical axis direction of the vehicle can be determined by the projection point in the image, denoted as a normalized vector $d_{V}^{\left(C\right)}$, and the transverse axis direction $d_{T}^{\left(C\right)}$ of the vehicle is the vector cross product of $d_{L}^{\left(C\right)}$, $d_{V}^{\left(C\right)}$. Therefore, the rotation matrix from the camera to the vehicle is $R^{\left(\mathrm{VC}\right)}={\left[{\left(d_{L}^{\left(C\right)}\right)}^{T}\;\;\;\;{\left(d_{V}^{\left(C\right)}\right)}^{T}\;\;\;\;{\left(d_{T}^{\left(C\right)}\right)}^{T}\right]}^{T}$.

### 2.3. SI-Line-Space Determination of Vehicle Translation

The center coordinates of the two ellipses in the image are $p_{C1}^{\left(I\right)}$, $p_{C2}^{\left(I\right)}$ and are adopted to determine the vehicle position. The camera normalization plane refers to the imaging plane located at *Z*^(C)^ = 1 in the camera coordinate system of the camera model, which is used to back-project pixels into normalized direction vectors through the inverse intrinsic parameter matrix K^−1^. $Q_{C1}=K^{-1}{\left(u_{1}^{\left(I\right)},v_{1}^{\left(I\right)},1\right)}^{T}$, $Q_{C2}=K^{-1}{\left(u_{2}^{\left(I\right)},v_{2}^{\left(I\right)},1\right)}^{T}$ are the directional vectors of the center points of two ellipses on the camera normalization plane. The center coordinates of the two ellipses on the camera normalization plane satisfy [28]

(9) $$p_{Ci}^{\left(C\right)}=s_{i}Q_{Ci}$$

where $p_{Ci}^{\left(C\right)}$ is the three-dimensional coordinate of the wheel center. *s_i_* is the scale factor from the camera normalization plane to the camera coordinate system.

As *s*_1_**Q**_C1_ and *s*_2_**Q**_C2_ in Equation (9) represent the three-dimensional positions of two wheel centers in the camera coordinate system, the vector connecting the two centers forms a vector with a length of the vehicle wheelbase *L*. Therefore, according to the rotation matrix $R^{\left(\mathrm{VC}\right)}$ from the vehicle coordinate system to the camera coordinate system, and the vector subtraction, in the camera coordinate system,

(10) $$s_{2}Q_{C2}^{\left(C\right)}-s_{1}Q_{C1}^{\left(C\right)}={\left(R^{\left(\mathrm{VC}\right)}\right)}^{-1}{\left(L\;\;0\;\;0\right)}^{T}$$

According to Equations (9) and (10),

(11) As=b

where $A=(Q_{C2}^{\left(C\right)}\text{-}Q_{C1}^{\left(C\right)})$, **s** = (*s*_2_ *s*_1_)^T^, **b** = (R^(VC)^)^−1^(*L* 0 0)^T^. The solution of the non-homogeneous linear system of equations is **s** = (A^T^A)^−1^A^T^**b**.

According to the camera imaging model, the relationship among the depth *Z_i_* of the wheel rim center, the rim circle diameter *D*, and the length of the elliptical major axis *a_i_* in the image is *Z_i_* = *Df*/*a_i_*, where *f* is the focal length. As *k*_11_ and *k*_22_ in the intrinsic parameter matrix K represent the equivalent focal lengths in the horizontal and vertical directions of the image, the focal length *f* is represented by (*k*_11_*k*_22_)^1/2^. Therefore, according to the camera pinhole model [29], the depth of the wheel-rim circle center in the coordinate system of the camera is

(12) $$Z_{i}=D{\left(k_{11}k_{22}\right)}^{1/2}/a_{i}$$

where *D* is the diameter of the wheel rim. *a_i_* is the major axis length of the projection ellipse.

According to Equations (11) and (12), the ratio factor between the real physical scale and the relative scale is $\alpha =\tilde{Z}/s_{1}$, where $\tilde{Z}=(Z_{1}+Z_{2})/2$. Thus, the true translation vector from the vehicle to the camera is scaled by the ratio factor *α* and solved by

(13) $$t^{\left(\mathrm{VC}\right)}=-\alpha Q_{C1}^{\left(C\right)}$$

**Figure 4:**
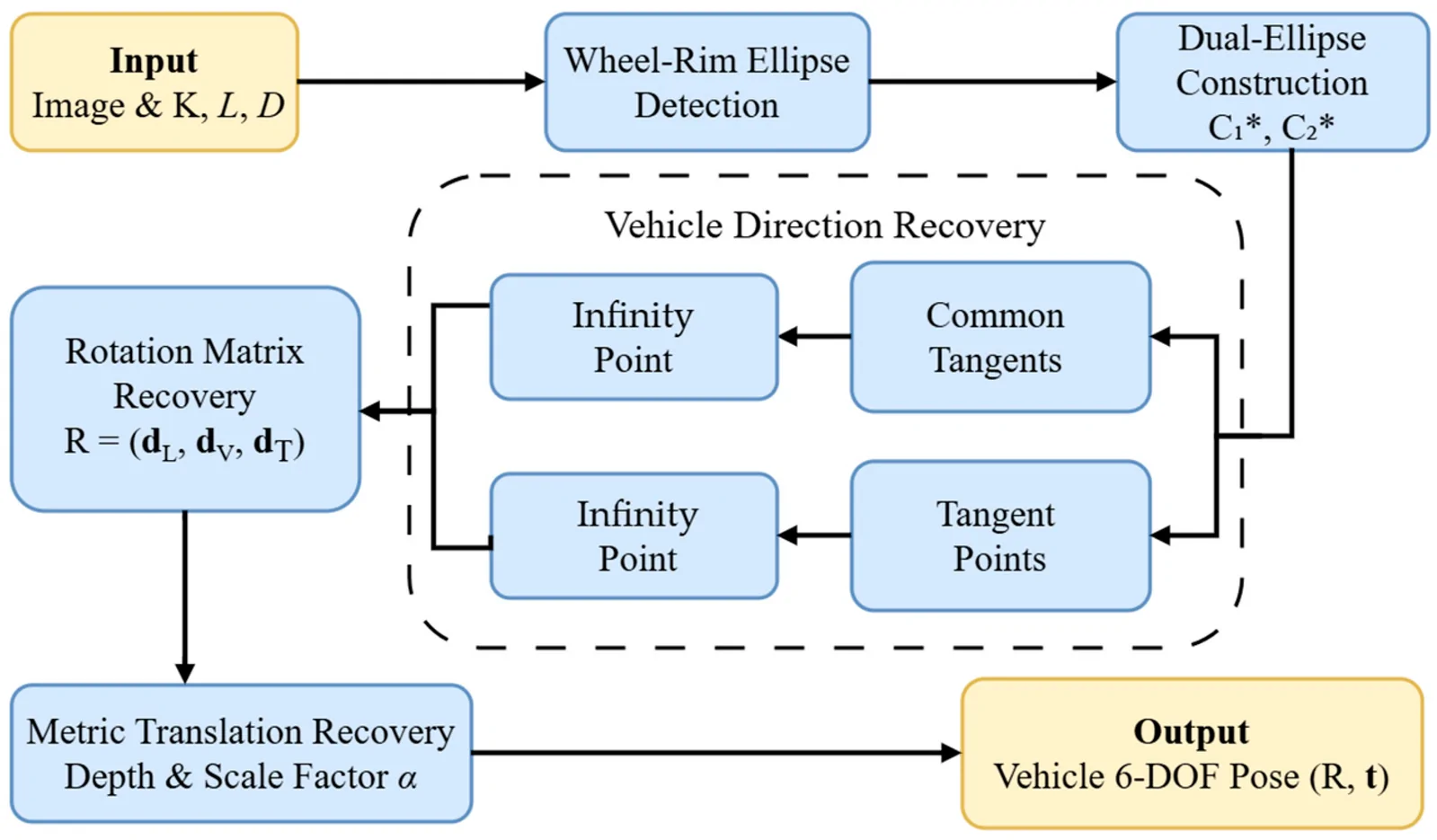
Vehicle pose measurement pipeline.

Therefore, the vehicle 6-DOF pose can be determined by the rotation and the translation from the vehicle to the camera. As the solution of the main steps such as generalized eigenvalues and vector cross products only involves fixed-dimensional 3 × 3 matrix operations, it can be regarded as a constant complexity O(1). The pipeline of the proposed method is summarized in Figure 4.

## 3. Measurement Experiment

### 3.1. Experimental Configuration

**Figure 5:**
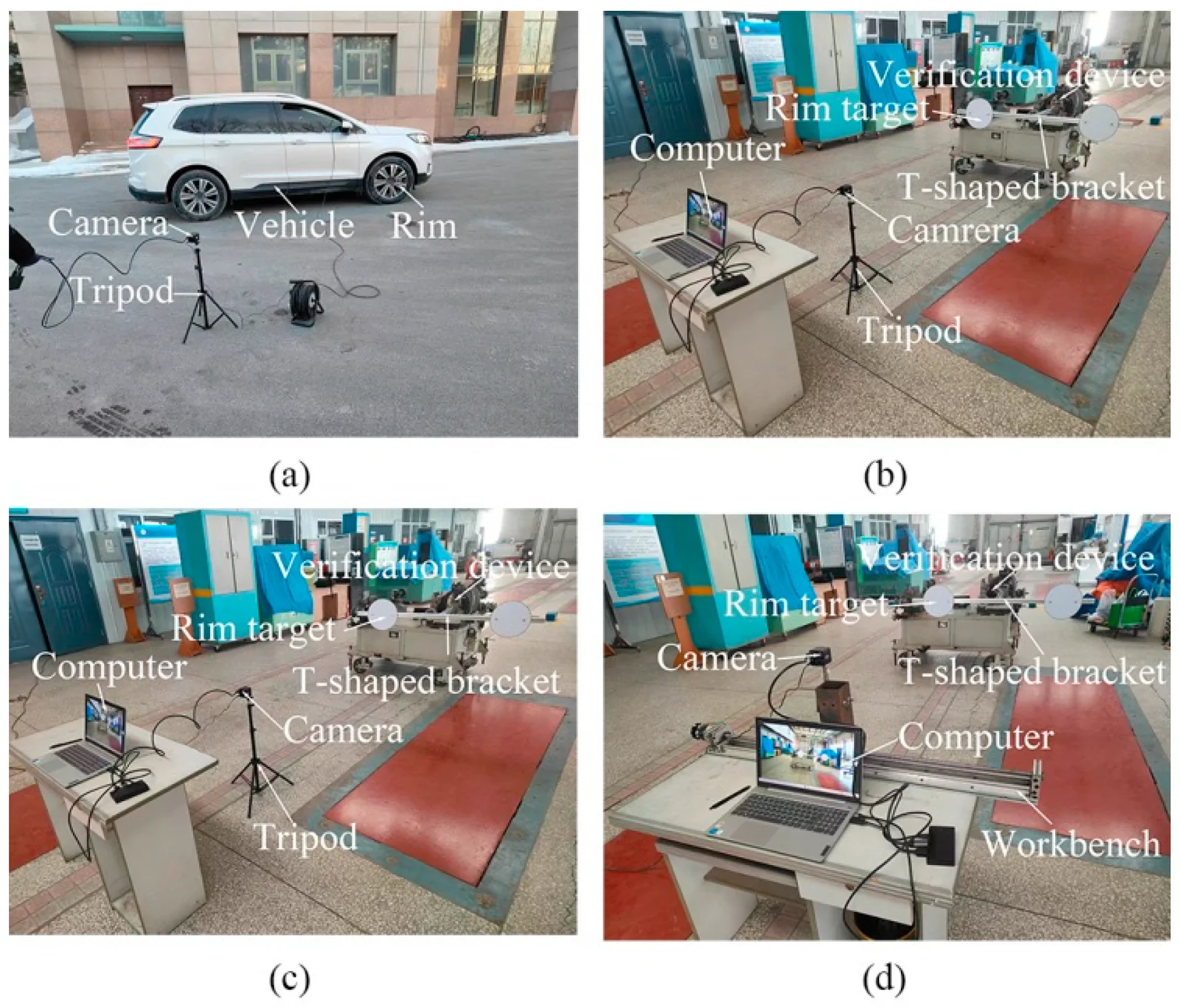
Experiments of vehicle pose measurement. (**a**) Vehicle pose measurement experiment. (**b**) Angle verification experiment. The rim target is located in the initial position. (**c**) Angle verification experiment. The rim target rotates around the vertical axis. (**d**) Translation verification experiment.

In order to evaluate the proposed vehicle pose measurement with the double-dual-ellipse feature of wheel rims, systematic experiments are conducted, specifically including simulation and comparative experiments, vehicle experiments, and verification experiments. The experimental devices are mainly composed of a camera, a tripod, two rim targets, a T-shaped bracket, a three-degree-of-freedom angle verification device, a linear displacement workbench, and the tested vehicle. The experimental instrumentation is shown in Figure 5. The vehicle pose measurement system composed of the camera and the tripod is shown in Figure 5a. Then, the two circular rim targets are fixedly connected to the three-degree-of-freedom angle verification device through the T-shaped bracket. The three-degree-of-freedom angle measurement results of the vehicle pose measurement system are verified according to the high-precision physical benchmarks generated by the three-degree-of-freedom angle verification device, as shown in Figure 5b,c. Moreover, the translation measurement results are verified by the linear displacement workbench, as shown in Figure 5d. The image resolution is 10,368 × 5888 pixels. The proposed closed-form pose solution requires 0.11 s/frame for the core pose solution. The total processing time is 10.76 s/frame, covering the image processing. Accordingly, the proposed method is currently intended for offline pose measurement on vehicle inspection lines. The YLR-J5 camera and a computer with a 2.4 GHz CPU and 16 GB RAM are used in the experiment. The ellipse detection method in Ref. [26] is employed to extract the wheel-related elliptical features from the images. This method is capable of extracting both wheel ellipses based on the complete ellipse edge structure rather than local discrete points.

### 3.2. Simulation and Comparative Experiment

**Figure 6:**
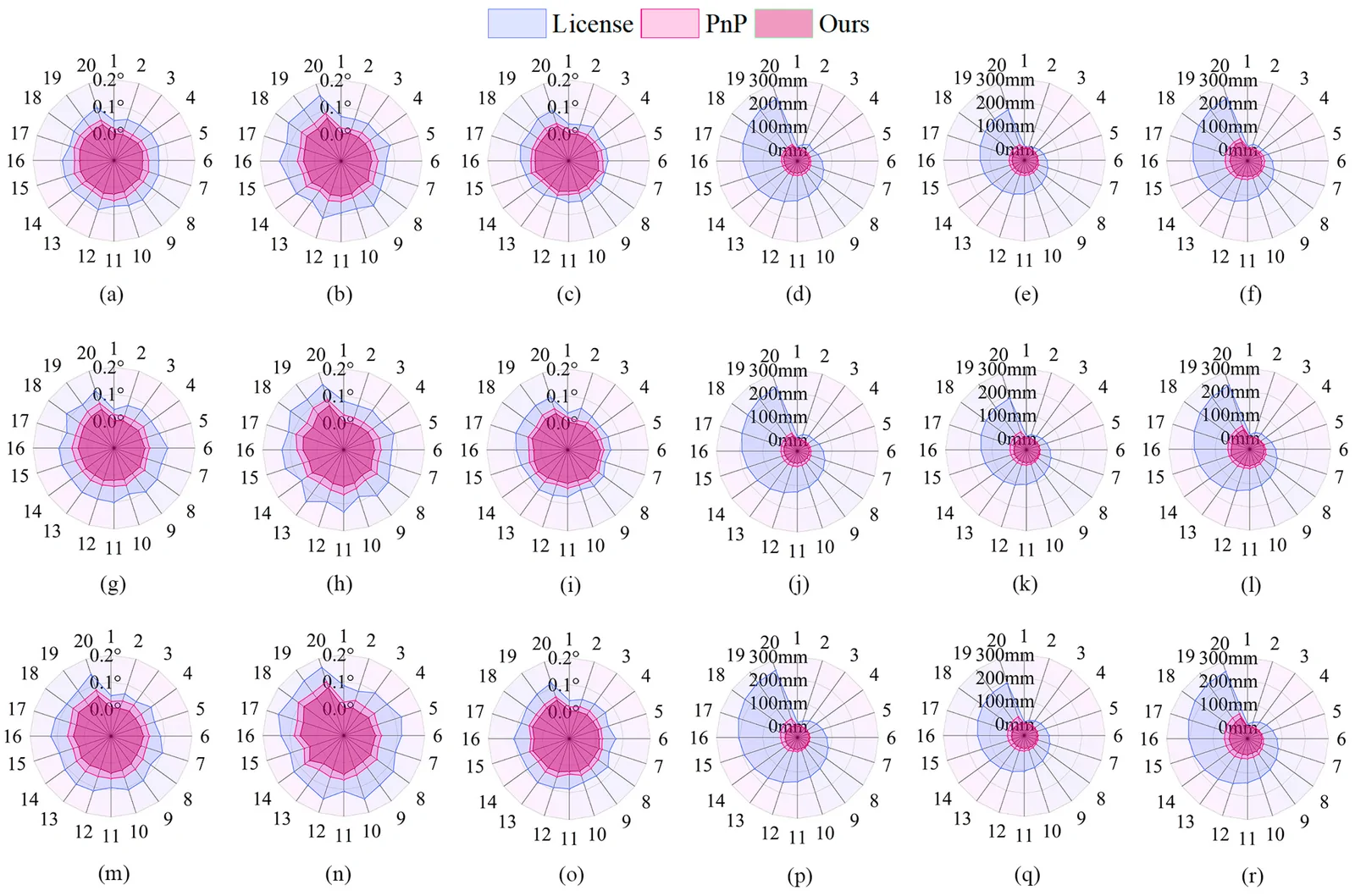
Simulation and comparative experiment results. (**a**–**c**) Rotation error at a distance of 2600 mm. (**d**–**f**) Translation error at a distance of 2600 mm. (**g**–**i**) Rotation error at a distance of 2800 mm. (**j**–**l**) Translation error at a distance of 2800 mm. (**m**–**o**) Rotation error at a distance of 3000 mm. (**p**–**r**) Translation error at a distance of 3000 mm.

For the purpose of studying the theoretical accuracy of the vehicle pose measurement method, simulation experiments are used to evaluate the accuracy of the 6 DOF of the vehicle pose measurement. Firstly, two rim circles are generated in the simulation vehicle coordinate system. Secondly, the two rim circles are rotated around the three coordinate axes of the simulation vehicle coordinate system at different angles from −10° to 10° with an interval of 1°, and projected into two ellipses on the image. Then, the two wheel rims are projected as two ellipses on the image by translating the camera along the three coordinate axes of the camera coordinate system. The translation distance ranges from −500 mm to 500 mm, with a step size of 50 mm. Finally, according to the two blurred image ellipses and the pose measurement method, the 6-DOF pose values of the vehicle are solved and compared with the previously set rotation and translation values to evaluate the accuracy of the measurement method. To investigate the influence of the detection distance on the measurement accuracy, the distances between the camera and the rim circles are set to 2600 mm, 2800 mm, and 3000 mm, respectively. The maximum and minimum errors of the measured 6 DOF are statistically analyzed, and the comparative experimental results with the license-plate-based rectangle method, the vanishing-point method in Ref. [30], and the classical geometric P*n*P method [31] are shown in Figure 6. All methods are evaluated under the same camera configuration, image resolution, detection distances, and vehicle pose settings.

The error results of rotation angles at different detection distances indicate that the rotation error along the three axes gradually increases with the increase in detection distance. For the case of rotation around the *O*^(V)^-*X*^(V)^ axis, at a detection distance of 2600 mm, the minimum rotation error of this method is 0.0003°, compared with 0.0476° and 0.0175° obtained by the two comparative methods, respectively. When the detection distance increases to 3000 mm, the maximum rotation error of this method is 0.0558°, which is lower than the corresponding errors of 0.1409° and 0.0788° from the two comparative methods. For the case of the rotation around the *O*^(V)^-*Y*^(V)^ axis, at a distance of

2600 mm, the minimum error of this method is 0.0016°, while the two comparative methods achieve errors of 0.0651° and 0.0223°, respectively. At a distance of 3000 mm, the maximum error of this method increases to 0.0867°, which remains lower than the errors of 0.1655° and 0.1096° achieved by the comparative methods. In the case of the rotation around the *O*^(V)^-*Z*^(V)^ axis, the minimum error occurs at a distance of 2600 mm. This method is 0.0005°, whereas the two comparative methods obtain errors of 0.0366° and 0.0169°, respectively. At the distance of 3000 mm, the maximum error of this method is 0.0501°, while the corresponding errors of the comparative methods increase to 0.1174° and 0.0630°. Overall, the translation errors along the three coordinate axes gradually increase with the increase in the detection distance. In terms of the translation along the *O*^(C)^-*X*^(C)^ axis, at the detection distance of 2600 mm, the minimum translation error of this method is 0.07 mm, which is lower than the errors of 12.70 mm and 3.39 mm obtained by the two comparative methods, respectively. When the distance increases to 3000 mm, the maximum translation error of this method is 6.14 mm, which is also lower than the corresponding errors of 255.75 mm and 35.97 mm. In the case of the translation along the *O*^(C)^-*Y*^(C)^ axis, the minimum translation error of this method at 2600 mm is 1.74 mm, while the two comparative methods produce errors of 9.47 mm and 5.68 mm, respectively. As the distance increases to 3000 mm, the maximum error of this method rises to 6.57 mm, whereas the errors of the comparative methods reach 189.49 mm and 35.29 mm. For the translation along the *O*^(C)^-*Z*^(C)^ axis, the minimum translation error of this method is 1.63 mm at 2600 mm, compared with 12.78 mm and 7.88 mm from the two comparative methods, respectively. At 3000 mm, the maximum translation error of this method is 44.18 mm, while the corresponding errors of the comparative methods increase to 255.65 mm and 63.48 mm, respectively. The average error, RMSE, and standard deviation of the 6 DOF at each distance are shown in Table 2. Therefore, at different detection distances, this method outperforms the comparative methods in terms of the rotation and the translation measurement accuracy.

**Figure 7:**
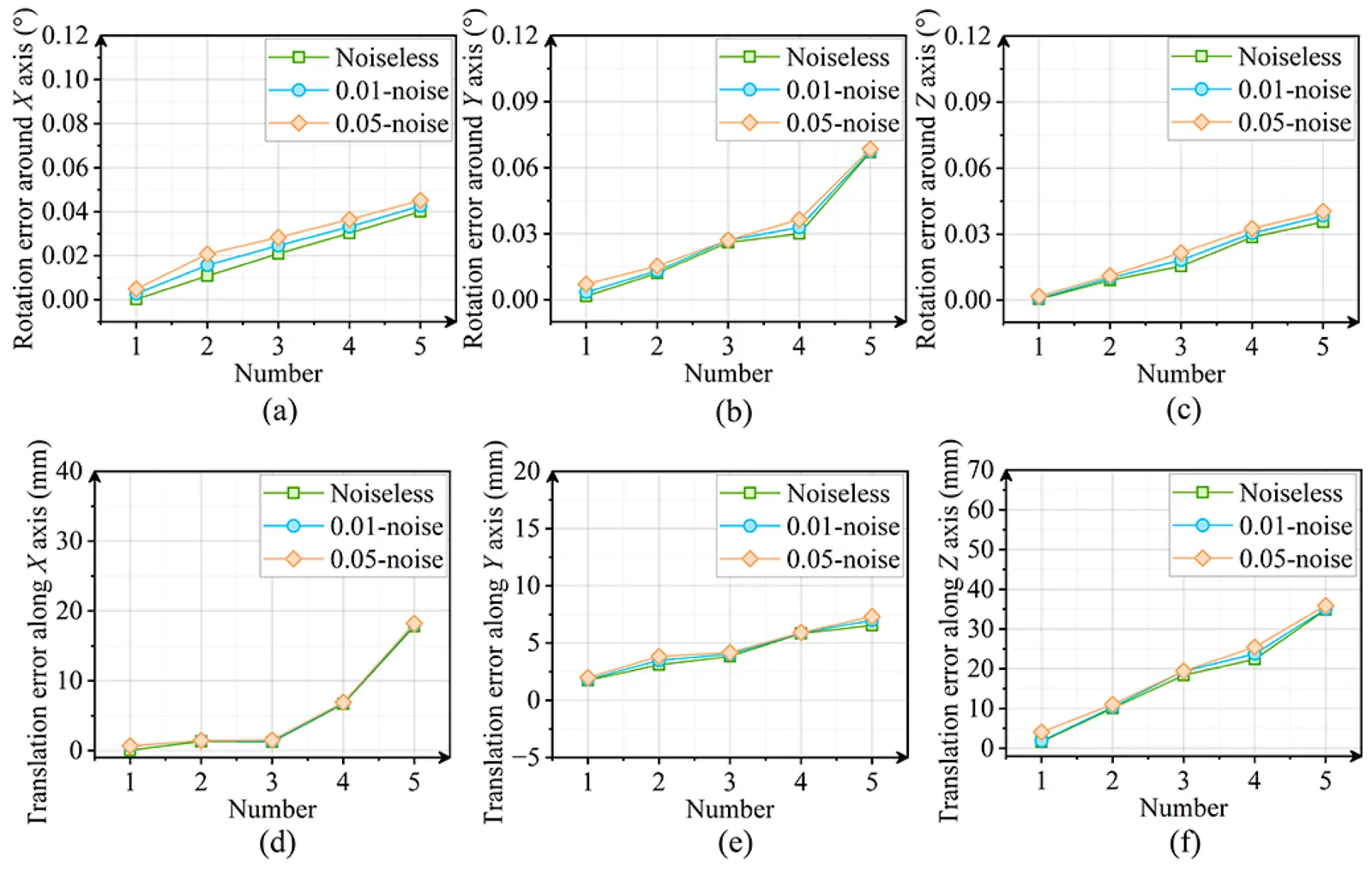
Rotation and translation errors under noise with standard deviations of 0, 0.01 pixels, and 0.05 pixels. (**a**–**c**) Rotation error around the *X*, *Y*, and *Z* axes, respectively. (**d**–**f**) Translation error along the *X*, *Y*, and *Z* axes, respectively.

To verify the robustness of the method, simulation experiments are conducted to test the error propagation from ellipse fitting to rotation and translation estimation. Gaussian noise with standard deviations of 0.01 pixels and 0.05 pixels is added to image pixels for error propagation testing, and the experimental results are shown in Figure 7. The influence of ellipse fitting deviation on the measurement error of vehicle rotation is within 0.02°, and the influence on the translation measurement error is within 2 mm. Therefore, the method is robust to small ellipse fitting deviations.

### 3.3. Vehicle Experiment

**Figure 8:**
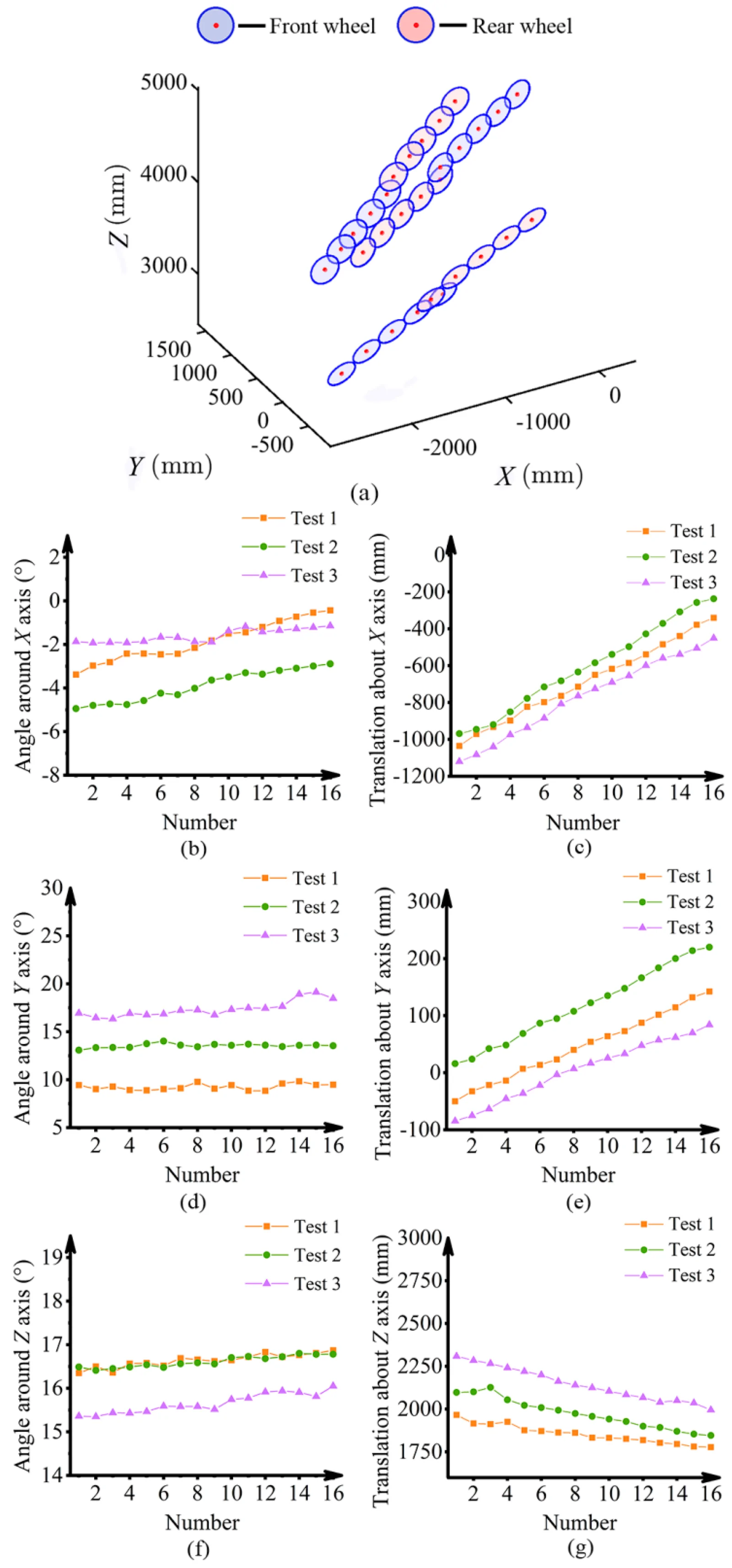
Measurement results of 6 DOF of the vehicle. (**a**) Motion trajectory. (**b**–**d**) Rotation around the *O*^(C)^-*X*^(C)^, *O*^(C)^-*Y*^(C)^, and *O*^(C)^-*Z*^(C)^ axes, respectively. (**e**–**g**) Translation about the *O*^(C)^-*X*^(C)^, *O*^(C)^-*Y*^(C)^, and *O*^(C)^-*Z*^(C)^ axes, respectively.

For the experimental evaluation of its practical applicability and measurement stability, the proposed method is tested in the vehicle experiment to measure the vehicle’s 6 DOF. The vehicle experiment is shown in Figure 5a. The experiment is performed on flat ground. The application scenario of this study is a controlled vehicle inspection scenario. The camera is fixed in position during one experiment and the experiment is performed three times at different camera positions. The experimental results of the 6 DOF of the tested vehicle are shown in Figure 8. The experiments indicate that the measurement results of the 6 DOF of the vehicle are stable. It can be seen that the three degrees of freedom representing rotation fluctuate smoothly. The angles between the three axes of the vehicle and the camera coordinate systems change little, so the vehicle maintains translational motion during driving. From Figure 8, it can also be seen that the *O*^(C)^-*X*^(C)^ component of the translation vector increases with the vehicle driving distance, indicating that the vehicle is moving forward and the *O*^(C)^-*X*^(C)^ is the main direction of the motion. Due to the angles between the coordinate axes of the vehicle coordinate system and the camera coordinate system, the vehicle moves further away from the camera in the *O*^(C)^-*Y*^(C)^ direction while it moves closer to the camera in the *O*^(C)^-*X*^(C)^ and *O*^(C)^-*Z*^(C)^ axes directions. The experimental results effectively verified that this method has stable 6-DOF measurement capabilities in the actual vehicle scenario, and the obtained motion trajectory is consistent with the real driving mode and spatial pose of the vehicle.

### 3.4. Verification Experiment

**Figure 9:**
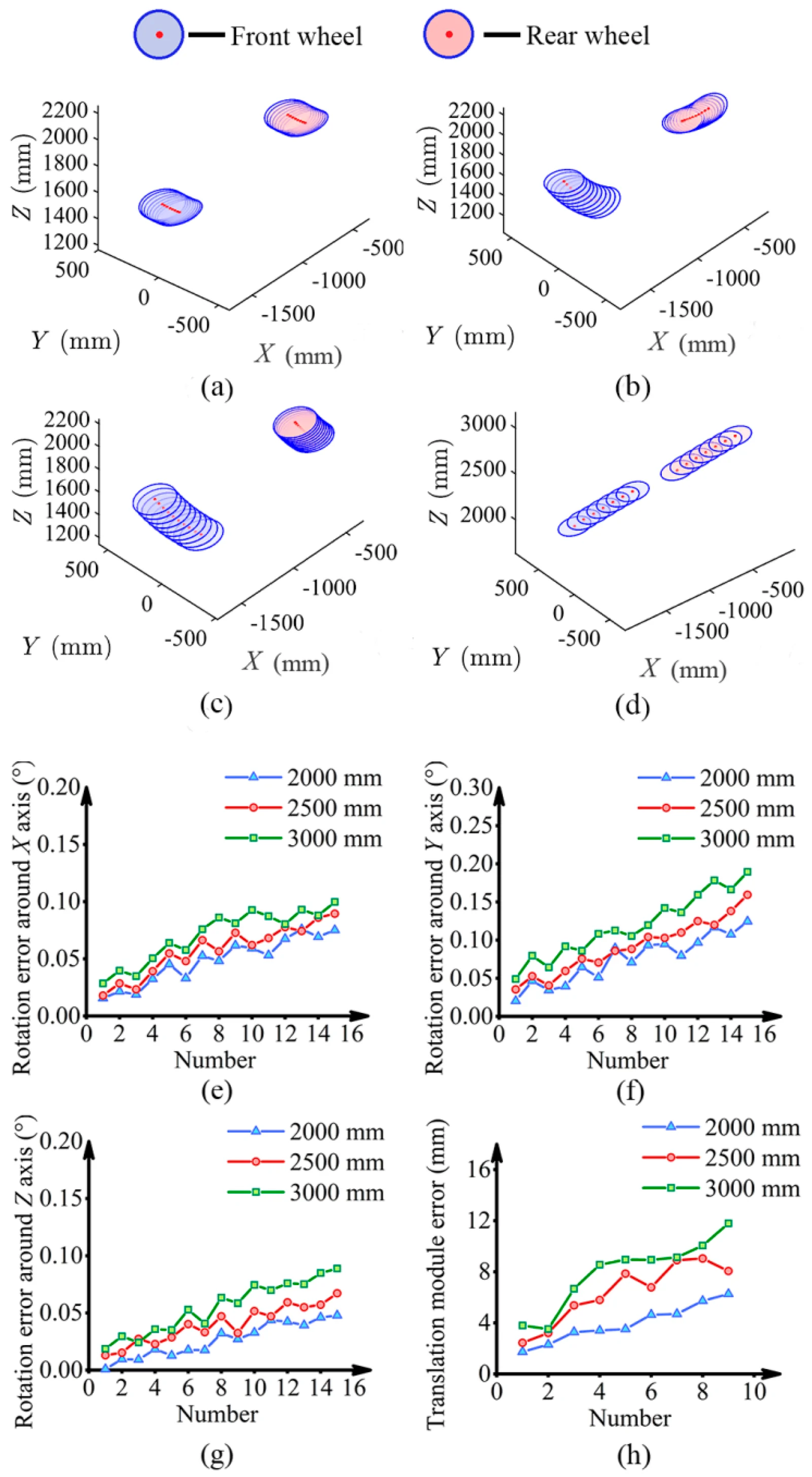
Verification results of pose measurement of the vehicle. (**a**–**c**) Motion trajectories of rotation around the *O*^(C)^-*X*^(C)^, *O*^(C)^-*Y*^(C)^, *O*^(C)^-*Z*^(C)^ axes, respectively. (**d**) Motion trajectory of translation. (**e**–**g**) Rotation errors around the *O*^(C)^-*X*^(C)^, *O*^(C)^-*Y*^(C)^, and *O*^(C)^-*Z*^(C)^ axes, respectively. (**h**) Translation errors.

To further verify the measurement accuracy of the method in a physical environment, physical benchmark experiments utilizing verification devices are conducted, as shown in Figure 5b–d. The accuracy of the physical benchmark equipment is ±30. In the rotation angle verification experiment, the wheel rim targets rotate around the three coordinate axes of the verification device coordinate system, from 0° to 15°, with an interval of 1°. In the translation experiment, the wheel rim targets are fixed and the camera is moved by the linear displacement workbench, ranging from 0 mm to 350 mm with an interval of 25 mm. In order to investigate the influence of the detection distance, the detection distances between the camera and the wheel rim targets are 2000 mm, 2500 mm, and 3000 mm, respectively. The experimental results of rotation at different distances are shown in Figure 9a–c,e,g. The experimental results show that the rotation error along the *O*^(C)^-*X*^(C)^, *O*^(C)^-*Y*^(C)^, and *O*^(C)^-*Z*^(C)^ axes gradually increases with the increase in the detection distance. When the wheel rim targets rotate around the *O*^(C)^-*X*^(C)^ axis, the minimum error of 0.0156° occurs at the detection distance of 2000 mm, while the maximum error of 0.0997° occurs at the detection distance of 3000 mm. For the case of rotation around the *O*^(C)^-*Y*^(C)^ axis, the minimum error is 0.0202° at 2000 mm, and the maximum error is 0.1893° at 3000 mm. When the rim targets rotate around the *O*^(C)^-*Z*^(C)^ axis, the detection distance of 2000 mm corresponds to the minimum error of 0.0008°, and the distance of 3000 mm corresponds to the maximum error of 0.0888°. The error results of the translation vector modulus at different detection distances are shown in Figure 9d,h. The error of the translation vector modulus gradually increases with the increase in the detection distance. At the detection distance of 2000 mm, the translation error ranges from 1.74 mm to 6.27 mm. When the distance increases to 2500 mm, the error range expands to 2.44 mm to 8.06 mm. For the detection distance of 3000 mm, the error further increases, with the minimum value of 3.52 mm and the maximum value of 11.79 mm. The average error, RMSE, and standard deviation at each distance are shown in Table 3. In summary, the verification results with the physical benchmark have validated the accuracy and effectiveness of this method in traceable physical environments.

## 4. Conclusions

In this study, a single-image vehicle pose measurement method based on the double-dual-ellipse feature of the wheel rims is proposed. Through the line space dual quadratic curve modeling, the Lagrangian optimization, and the generalized eigenvalue modeling, the analytical solution of the common tangent of the wheel rim ellipse is achieved, and the vehicle rotation matrix is directly solved by the intersection points of the common tangent. Moreover, the method establishes a closed-form solution model with the vehicle wheelbase for the scale factor and translation vector, which can restore the 6-DOF pose of the vehicle with only a SI. Systematic experimental verification shows that the method exhibits high measurement accuracy. In the physical benchmark experiment, the measurement error of the rotation angle is less than 0.19°, and the translation error is less than 11.79 mm at the detection distance of 3000 mm. It also maintains stable and reliable performance under different distance conditions. In terms of limitations of the method, it assumes that the front and rear wheels should be circular. Although the ellipse extraction method in [26] is designed to be robust to image noise and edge discontinuities, severe wheel occlusion may leave insufficient visible rim contours for reliable ellipse fitting, and extreme pitch angles should be avoided to prevent the degradation of projected rim ellipses. This study provides a single-image vehicle pose measurement method for quasi-static and offline vehicle inspection applications. Under the vehicle inspection line conditions, the proposed method provides a stable geometric solution for vehicle pose measurement.

## Figures and Tables

**Table 1:** List of abbreviations.

Abbreviation	Full Term
DOF	Degrees of Freedom
6-DOF	Six Degrees of Freedom
SI	Single Image
CNN	Convolutional Neural Network
P*n*P	Perspective-*n*-Point
RGB	Red, Green, and Blue

**Table 2:** Evaluation indices of simulation experiment.

Index	Distance	Rot-*x* (°)	Rot-*y* (°)	Rot-*z* (°)	Trans-*x* (mm)	Trans-*y* (mm)	Trans-*z* (mm)
Average Error	2600	0.0192	0.0263	0.0160	2.25	3.25	18.67
	2800	0.0214	0.0290	0.0232	2.75	4.63	19.14
	3000	0.0271	0.0341	0.0242	2.98	5.05	20.45
RMSE	2600	0.0228	0.0314	0.0186	2.75	3.45	21.00
	2800	0.0250	0.0353	0.0258	3.74	4.79	22.35
	3000	0.0303	0.0429	0.0278	3.66	5.17	23.26
STD	2600	0.0117	0.0172	0.0090	1.59	1.08	9.64
	2800	0.0129	0.0201	0.0112	2.53	1.17	11.07
	3000	0.0136	0.0254	0.0137	2.13	1.25	11.55

**Table 3:** Evaluation indices of verification experiment.

Index	Distance	Rot-*x* (°)	Rot-*y* (°)	Rot-*z* (°)	Trans-*x* (mm)
Average error	2000	0.0486	0.0753	0.0283	3.95
	2500	0.0576	0.0912	0.0398	6.39
	3000	0.0706	0.1192	0.0551	7.94
RMSE	2000	0.0524	0.0813	0.0304	4.20
	2500	0.0615	0.0977	0.0595	6.77
	3000	0.0740	0.1259	0.0595	8.35
STD	2000	0.0196	0.0307	0.0136	1.42
	2500	0.0215	0.0350	0.0161	2.25
	3000	0.0224	0.0407	0.0223	2.61

## Data Availability

Data is contained within the article.
